# An oral recombinant *Salmonella enterica *serovar Typhimurium mutant elicits systemic antigen-specific CD8+ T cell cytokine responses in mice

**DOI:** 10.1186/1757-4749-1-9

**Published:** 2009-04-29

**Authors:** Nyasha Chin'ombe, William R Bourn, Anna-Lise Williamson, Enid G Shephard

**Affiliations:** 1 Institute of Infectious Disease and Molecular Medicine, Faculty of Health Sciences, University of Cape Town, Anzio Rd, Observatory 7925, Cape Town, South Africa; 2 Kapa Biosystems (Pty) Ltd, Observatory 7925, Cape Town, South Africa; 3 MRC/UCT Liver Research Centre, Department of Medicine, Faculty of Health Sciences, University of Cape Town, Anzio Rd, Observatory 7925, Cape Town, South Africa

## Abstract

**Background:**

The induction of antigen-specific CD8+ T cell cytokine responses against an attenuated, oral recombinant *Salmonella enterica *serovar Typhimurium vaccine expressing a green fluorescent protein (GFP) model antigen was investigated. A GFP expression plasmid was constructed in which the *gfp *gene was fused in-frame with the 5' domain of the *Escherichia coli β*-galactosidase *α*-gene fragment with expression under the *lac *promoter. Groups of mice were orally immunized three times with the bacteria and systemic CD8+ T cell cytokine responses were evaluated.

**Results:**

High level of the GFP model antigen was expressed by the recombinant *Salmonella *vaccine vector. Systemic GFP-specific CD8+ T cell cytokine (IFN-γ and IL-4) immune responses were detected after mice were orally vaccinated with the bacteria. It was shown that 226 net IFN-γ and 132 net IL-4 GFP-specific SFUs/10e6 splenocytes were formed in an ELISPOT assay. The level of IFN-γ produced by GFP peptide-stimulated cells was 65.2-fold above background (p < 0.05). The level of IL-4 produced by the cells was 10.4-fold above background (p < 0.05).

**Conclusion:**

These results suggested that a high expressing recombinant *Salmonella *vaccine given orally to mice would elicit antigen-specific CD8+ T cell responses in the spleen. *Salmonella *bacteria may, therefore, be used as potential mucosal vaccine vectors.

## Background

Most *Salmonella *bacteria invade their hosts (human or animal) via the mucosal route to cause systemic infection [1]. They are taken up by phagocytes and they stay in the phagosomes of these cells. Antigens from *Salmonella *are mainly targeted to the MHC class II presentation pathway for induction of CD4+ T cell immune responses. However, both CD4+ and CD8+ T lymphocytes are crucial for protective immune responses against intracellular pathogens such as *Salmonella *[2-4]. In recent years, attenuated strains of *Salmonella *have been explored as potential mucosal vaccine vectors for heterologous antigens [5-11]. One of the main advantages of using *Salmonella *as vaccine vectors is their ability to induce both mucosal and systemic immune responses to the foreign antigens. In order to investigate the induction of antigen-specific CD8+ T cell responses to a foreign antigen, we developed a recombinant *Salmonella *vector expressing jellyfish *Aequorea victoria *green fluorescent protein (GFP) as a model antigen. The GFP model antigen contains a mouse H-2K^d^-restricted class I epitope, HYLSTQSAL, identified previously by Gambotto and co-workers [12] and can be used to evaluate CD8+ T cell responses after vaccinations. We then investigated the potential of using a *Salmonella *vaccine in delivering the GFP CD8+ epitope to the immune system. The study was done against a backdrop for the need to develop vaccines that induce CD8+ T cell responses in the mucosal and systemic compartments in which *Salmonella *may be used as a mucosal vector administered orally. In order to understand the steps required for the development of such vaccines, we therefore constructed the recombinant *Salmonella enterica *serovar Typhimurium expressing GFP as a model foreign antigen and tested its systemic immune responses in mice after oral vaccination by gavage.

## Results

### A recombinant *Salmonella *vaccine vector was constructed

**Figure 1:**
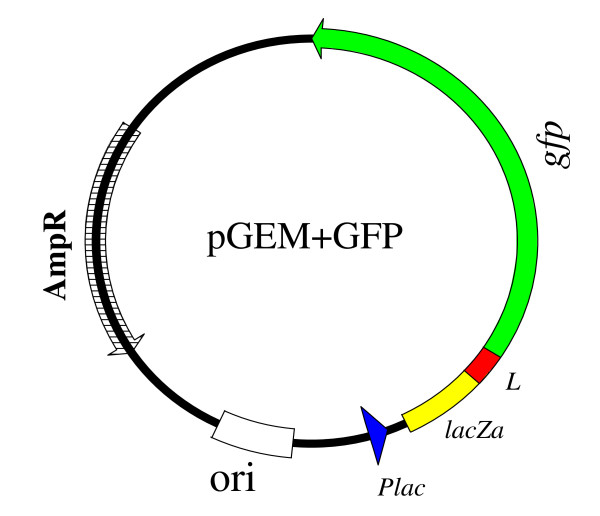
**The GFP expression plasmid (pGEM+GFP)**. The *gfp *was fused in-frame to the *β*-galactosidase *α*-gene in pGEM-Teasy plasmid. A small linker (L) was included (in-frame) between the *gfp *and *β*-galactosidase *α*-gene (*lacZa*). *E. coli lac *(*lactose*) promoter was upstream the genes. A start codon was in the *β*-galactosidase *α*-gene and a stop codon was included at the end of the *gfp *gene. The expression cassette contained an *E. coli *origin of replication (ori) and ampicillin resistance gene (AmpR).

A prokaryotic expression cassette was developed in which the *gfp *gene was fused in-frame with an *E coli β*-galactosidase *α*-fragment sequence (N-terminus) (Figure 1). The *gfp *gene was amplified and cloned into pGEM-Teasy plasmid vector. The *β*-galactosidase *α*-fragment with DNA sequence (5'-ATG ACC ATG ATT ACG CCA AGC TAT TTA GGT GAC ACT ATA GAA TAC TCA AGC TAT GCA TCC AAC GCG TTG GGA GCT CTC CCA TAT GGT CGA CCT GCA GGC GGC CGC GAA TTC ACT AGT GAT-3') had 24 amino acids (MTMITPSYLG DTIEYSSYAS NALGALPYGR PAGGREFTSD) and the peptide was 4.2 kDa in size. A small linker (**L**) sequence with 15 codons (5-TAT GGC GCC AAA GAC TCC GGC TCC GCC GGT TCC GCC GGC TCA GCT-3) was incorporated between the *β*-galactosidase *α*-fragment and *gfp*. The linker peptide had 15 amino acids (YGAKDSGSAG SAGSA) and a molecular weight of 1.266 kDa. The *gfp *gene had 237 amino acids (SKGEELFTGV VPILVELDGD VNGHKFSVSG EGEGDATYGK LTLKFICTTG KLPVPWPTLV TTFSYGVQCF SRYPDHMKRH DFFKSAMPEG YVQERTISFK DDGNYKTRAE VKFEGDTLVN RIELKGIDFK EDGNILGHKL EYNYNSHNVY ITADKQKNGI KANFKIRHNI EDGSVQLADH YQQNTPIGDG PVLLPDNHYL STQSALSKDP NEKRDHMVLL EFVTAAGITH GMDELYK) and a molecular weight of 26.6 kDa. The GFP contains a Balb/C mouse CD8+ T cell epitope, HYLSTQSAL. The whole *β*-galactosidase-GFP fusion protein was 32.1 kDa. A preferred translation stop codon (TAAG) which was incorporated in the PCR primer, GR, was found at the end of the *gfp *gene. There was also an extra stop codon, TAAT, one codon downstream the end of the *gfp *gene.

**Figure 2:**
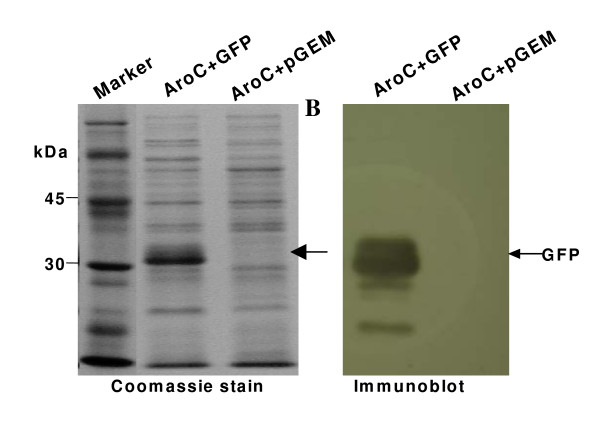
**GFP expression by the *Salmonella *vaccine vector**. Recombinant *Salmonella *expressing GFP (AroC+GFP) or *Salmonella *carrying an empty plasmid (AroC+pGEM) were grown overnight. GFP expression by the bacteria was determined by sodium dodecyl sulphate-polyacrylamide gel electrophoresis (A) and confirmed by Western Blotting (B).

Very high level constitutive expression of GFP antigen by the recombinant *Salmonella enterica *serovar Typhimurium, AroC+GFP, was demonstrated (Figure 2). Colonies and cultures of the bacterial vaccine, AroC+GFP, fluoresced brightly green under UV light. SDS-PAGE analysis showed that GFP antigen was the most highly expressed antigen by the *Salmonella *vaccine vector (Figure 2A). The GFP protein band was visible on the Coomassie-stained gel. Western blotting further confirmed that GFP antigen was expressed at very high levels by the vaccine vector (Figure 2B). There was no expression of GFP by the negative control vaccine, AroC+pGEM.

### Oral vaccination induces IFN-γ and IL-4 cytokine producing CD8+ splenocytes

The induction of GFP-specific CD8+ T cells in the spleen was evaluated on Day 84 after oral vaccination of mice with a dose of 10e8 colony-forming units either with AroC+GFP or the control (AroC+GEM) on Days 0, 28 and 56. Both IFN-γ and IL-4 producing GFP-specific CD8+ T cells were evaluated after sacrifice of mice. A high magnitude of GFP-specific CD8+ T cells was detected when the GFP peptide was included in the IFN-γ and IL-4 ELISPOT assays (Tables 1 and 2). The number of cells secreting IFN-γ after stimulation with a GFP CD8 peptide were significantly higher in AroC+GFP than in the negative control vaccine, AroC+pGEM (p < 0.05) (Table 1). There was no significant difference in response between the two groups when the cells were stimulated with media or full-length GFP (p > 0.05). Response to the LPS stimulation differed between the two groups (p < 0.05). Analysis of the responses within the AroC+GFP group showed that the number of cells producing IFN-γ were significantly higher when stimulated with GFP CD8 peptide than when not stimulated (p < 0.05) (Table 1). In the negative vaccine group, AroC+pGEM, there was no difference in response in GFP peptide-stimulated cells and unstimulated cells (p > 0.05) (Table 1).

**Table 1:** The magnitude of GFP-specific CD8+ T cell responses as measured by IFN-γ ELISPOT assay

**Stimulant**	**IFN-γ SFUs/10e6 cells**		**p-value**
		
	**AroC+GFP Group**	**AroC+pGEM Group**	
Media	6 ± 2	7 ± 5	0.84
GFP CD8 peptide	232 ± 15	6 ± 2	0.0001
GFP protein	4 ± 0	4 ± 2	1.00
*Salmonella *LPS	244 ± 39	375 ± 62	0.037

**Vaccine group**	**Media**	**GFP peptide**	**p-value**

AroC+GFP	6 ± 2	233 ± 15	0.0001
AroC+pGEM	7 ± 5	6 ± 2	0.70

Groups of mice were vaccinated three times (Days 0, 28 and 56) with live recombinant *Salmonella *vaccine expressing GFP (AroC+GFP) or a negative *Salmonella *control vaccine not expressing any antigen (AroC+pGEM). On Day 84 (28 days after the last inoculation), splenocytes from the sacrificed mice were incubated with media only (negative assay control), or stimulated with GFP CD8+ T cell peptide (HYLSTQSAL), full-length GFP or *Salmonella *LPS in an IFN-γ ELISPOT assay. The mean number of spots ± SD in triplicate wells was calculated and expressed as IFN-γ SFUs/10e6 cells. Difference in response between or within vaccines was determined. Responses differ significantly if the p-values are less than 0.05 and do not differ significantly if the p-values are greater than 0.05.

**Table 2:** The magnitude of GFP-specific CD8+ T cell responses as measured by IL-4 ELISPOT assay

**Stimulant**	**IL-4 SFUs/10e6 cells**		**p-value**
		
	**AroC+GFP Group**	**AroC+pGEM Group**	
Media	16 ± 10	12 ± 2	0.55
GFP CD8 peptide	148 ± 56	12 ± 13	0.015
GFP protein	18 ± 13	29 ± 26	0.56
*Salmonella *LPS	55 ± 29	26 ± 9	0.17

**Vaccine group**	**Media**	**GFP peptide**	**p-value**

AroC+GFP	16 ± 10	148 ± 56	0.016
AroC+pGEM	12 ± 2	12 ± 13	0.97

Groups of mice were vaccinated three times (Days 0, 28 and 56) with live recombinant *Salmonella *vaccine expressing GFP (AroC+GFP) or a negative *Salmonella *control vaccine not expressing any antigen (AroC+pGEM). On Day 84 (28 days after the last inoculation), splenocytes from the sacrificed mice were incubated with media only (negative assay control), or stimulated with GFP CD8+ T cell peptide (HYLSTQSAL), full-length GFP or *Salmonella *LPS in an IL-4 ELISPOT assay. The mean number of spots ± SD in triplicate wells was calculated and expressed as IL-4 SFUs/10e6 cells. Difference in response between or within vaccines was determined. Responses differ significantly if the p-values are less than 0.05 and do not differ significantly if the p-values are greater than 0.05.

The number of cells from AroC+GFP vaccine group producing IL-4 were also significantly higher than in AroC+pGEM after stimulation with GFP CD8 peptide (p < 0.05) (Table 2). However no significant difference in response between the two groups were observed when the cells were stimulated with media, full-length GFP or *Salmonella *LPS (p > 0.05). Within the AroC+GFP group, the number of cells producing IL-4 were significantly higher when stimulated with GFP CD8 peptide than when unstimulated (p < 0.05) (Table 2). No difference in IL-4 responses was observed within the AroC+pGEM group between GFP peptide-stimulated and unstimulated cells (p > 0.05) (Table 2).

**Figure 3:**
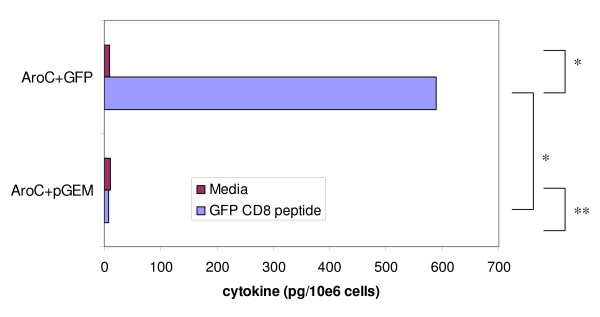
**The magnitude of GFP-specific CD8+ T cell responses as determined by quantification of IFN-γ cytokine**. Groups of mice were vaccinated three times (Days 0, 28 and 56) with live recombinant *Salmonella *vaccine expressing GFP (AroC+GFP) or a negative *Salmonella *control vaccine not expressing any antigen (AroC+pGEM). On Day 84 (28 days after the last inoculation), splenocytes from the sacrificed mice were incubated with media only (negative assay control) or stimulated with GFP CD8+ T cell peptide (HYLSTQSAL), and the amounts of IFN-γ measured by CBA assay. Each bar in the graphs represents the average picogram amount of cytokine produced per 10e6 splenocytes in 48 hrs of stimulation. One asterisk indicates values that differ significantly (p < 0.05). Two asterisks indicate values that do not differ significantly (p > 0.05).

**Figure 4:**
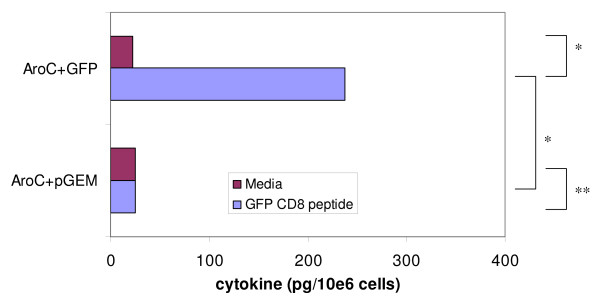
**The magnitude of GFP-specific CD8+ T cell responses as determined by quantification of IL-4 cytokine**. Groups of mice were vaccinated three times (Days 0, 28 and 56) with live recombinant *Salmonella *vaccine expressing GFP (AroC+GFP) or a negative *Salmonella *control vaccine not expressing any antigen (AroC+pGEM). On Day 84 (28 days after the last inoculation), splenocytes from the sacrificed mice were incubated with media only (negative assay control) or stimulated with GFP CD8+ T cell peptide (HYLSTQSAL), and the amounts of IL-4 measured by CBA assay. Each bar in the graphs represents the average picogram amount of cytokine produced per 10e6 splenocytes in 48 hrs of stimulation. One asterisk indicates values that differ significantly (p < 0.05). Two asterisks indicate values that do not differ significantly (p > 0.05).

The cytometric bead array (CBA) assay and flow cytometry analysis were used to quantify the IFN-γ and IL-4 simultaneously produced by splenocytes after stimulation with the GFP H-2K^d ^binding peptide (HYLSTQSAL). Cells from AroC+GFP produced higher levels of IFN-γ when stimulated with the GFP CD8 peptide than when unstimulated (p < 0.05) (Figure 3). The IFN-γ cytokine levels were also higher in the test group (AroC+GFP) than in the negative vaccine group (AroC+pGEM) (p < 0.05) (Figure 3). There was no difference in the amount of IFN-γ produced between stimulated and unstimulated cells within the negative control vaccine (p > 0.05) (Figure 3). As with IFN-γ, the same trends were observed with IL-4 (Figure 4). Cells from AroC+GFP produced higher levels of IL-4 when stimulated with GFP peptide than when unstimulated (p < 0.05). The level of the IL-4 produced by the stimulated cells was 10.4-fold above background. GFP peptide-stimulated cells from AroC+GFP also produced significantly higher levels of IL-4 than cells from the negative control vaccine, AroC+pGEM (p < 0.05). The results from CBA further confirmed the ELISPOT results which showed that a significant number of cells produced both IFN-γ and IL-4 after stimulation with the GFP CD8 peptide. Further analysis of the both ELISPOT and CBA assay results showed that GFP-specific IFN-γ was produced at a rate of 2.56 pg/cell as opposed to GFP-specific IL-4 which was produced at 1.63 pg/cell.

## Discussion

Attenuated *Salmonella *bacteria have the potential of being used as vaccine vectors for foreign antigens (5–11). One of the key challenges with these vaccine delivery systems is to optimize the expression of high levels of the foreign antigens for successful delivery to the immune system. In the current study, a strategy based on *E. coli lac *operon control sequences was employed and tested for expression of a model foreign antigen, *Aequorea victoria *green fluorescent protein, in *aro*C *Salmonella enterica *serovar Typhimurium vaccine mutant. The *E. coli lac *promoter was used and the *gfp *gene was successfully fused in-frame with first 40 codons of the *E coli β*-galactosidase *α*-fragment. The inclusion of the N-terminal domain of the *β*-galactosidase *α*-gene fragment, which itself is an *E. coli *bacterial peptide potentially contributed to the high-level expression of GFP observed in the *Salmonella enterica *serovar Typhimurium vector. Fusing foreign proteins to other prokaryotic peptides has the potential of enhancing the expression of the cloned genes [13]. Furthermore, fusion proteins have been shown to be, in most cases, resistant to proteolytic degradation, thereby overcoming the problems of instability normally associated with foreign proteins [14,15]. The fusion of *gfp *to the 5'-domain of LacZα also potentially stabilized GFP mRNA of the antigen gene and increased its half-life. In a similar study, it was shown that fusing genes to the 5' UTR (untranslated region) of *ompA *was effective in stabilizing the mRNA transcripts [16]. Other considerations that potentially contributed to the high level expression of GFP antigen were the nature of the ribosome-binding site, the origin of replication (ori), promoter (*lac*) properties, and translation termination sequences. These transcriptional and translational domains are present in the pGEM-Teasy plasmid (Promega, USA). The origin of replication of the pGEM-Teasy plasmid allowed for high copy number of the plasmid (300–400 copies per cell) in *Salmonella *vector, thereby increasing the *gfp *gene dosage and high expression of the antigen. The natural Shine Dalgarno sequence (ribosome binding site) for the *LacZα *gene in the pGEM-Teasy plasmid was used for efficient bacterial ribosome binding. The stop codon, TAA(G) was used in the pGEM+GFP plasmid to increase efficiency of translation termination. The high-level GFP expression was anticipated to facilitate the delivery of sufficient antigen to the immune system by the *Salmonella *vector after vaccination.

Development of bacterial vaccine vectors that provoke antigen-specific CD8+ T cell responses in the mucosal and systemic compartments is a key challenge. In this study, we were able to demonstrate that oral vaccination of mice with a recombinant *aro*C *Salmonella enterica *serovar Typhimurium mutant overexpressing a heterologous model antigen could induce antigen-specific CD8+ T cell cytokine immune responses in the spleen. Using the ELISPOT assay, it was shown that after three oral immunizations of mice with AroC+GFP, there was production of both antigen-specific IFN-γ and IL-4 cytokine secreting CD8+ cells in the spleen. The ELISPOT results were further confirmed by the CBA assay which showed that high levels of IFN-γ and IL-4 cytokines could be secreted by the splenocytes when stimulated with a GFP CD8 peptide. The induction of LPS-specific IFN-γ and IL-4 suggested that the bacterial vaccine was delivered successfully to the immune system.

*Salmonella *antigens or heterologous antigens expressed by *Salmonella *vaccine vectors are expected to be presented mainly by the MHC-II molecules to give predominantly antigen-specific CD4+ T cell responses. This is mainly because *Salmonella *bacteria always dwell in the phagosomes and antigens are presented to the immune system by the MHC Class II pathway. The mechanisms by which *Salmonella*-expressed antigens are presented to the immune system by the MHC Class I pathway to induce CD8+ T cell responses is still poorly understood. However, it is known that *Salmonella *have a high tropism for dendritic cells and these cells have the capacity of cross-priming exogenous antigens for induction of CD8+ T cell responses [17-22]. Dendritic cells can also engulf the *Salmonella*-infected apoptotic cells which may be a key source of antigens that can be processed for induction of CD8+ T cell responses [17,18]. It seems that the high-level expression of the GFP shown in this study may have facilitated antigen processing and cross-presentation for induction of CD8+ T cell responses. The high amounts of the antigen also potentially improved the immunodominance of GFP CD8+ epitope over *Salmonella *vector epitopes. It has been demonstrated that antigen abundance (antigen dose) is one of the crucial factors that determine CD8+ T cell immunodominance [23,24]. It was not clear whether IFN-γ or IL-4 cytokines observed in this study were secreted by the same or different CD8+ T cell populations as we did not do flow cytometry to determine this. The ELISPOT assays do not allow the characterization of the effector cell populations secreting the two cytokines. However, the data is only suggestive that IFN-γ was produced by type 1 CD8+ T (Tc1) cells while IL-4 was produced by type 2 CD8+ (Tc2) cells. The possible polarized pattern of secreted cytokines by CD8+ T cells against the GFP model antigen delivered by a *Salmonella *vaccine observed in the current study might have a great relevance to immune responses against many diseases. CD8+ Tc1 cells produce cytokines such as IFN-γ and TNF-*α *that are critical in prevention or control of infection. However, the immunological and clinical significance of CD8+ Tc2 cells is still poorly understood. Some reports suggest that that Tc2 cells provide B cell help by secretion of IL-4 and would display cytotoxicity function just like the Tc1 cells [25-27]. Tc2 cells may also be correlated with better antibody immune responses [28,29]. High numbers of CD8+ T cells (Tc2) producing IL-4, but not IFN-γ, have been found in AIDS patients [30]. It has also been established that Tc2 cells play a role in reducing metastasis of lung cancer [31]. Although a *Salmonella *vaccine vector eliciting foreign antigen-specific IFN-γ may be useful, the impact of a vaccine that induces antigen-specific IL-4 is poorly understood.

This study is unique in that we showed that expression of a foreign antigen in the bacterial cytoplasmic space could elicit antigen-specific cellular responses in vaccinated mice. Other studies have only shown that CD8+ T cell responses in mice could only be induced when antigens were secreted from the bacteria or when prime-boost regimens were used in the vaccination [33-36]. Unlike in most studies, we also looked at the simultaneous induction of both IFN-γ and IL-4 cytokine responses elicited in the systemic compartment of *Salmonella*-vaccinated mice.

## Conclusion

In conclusion, we have shown that an oral recombinant *Salmonella *mutant could be used as a vaccine vector that could deliver a GFP model antigen for induction of systemic antigen-specific CD8+ T cell cytokine (IFN-γ and IL-4) responses. Using the current study as a model, future investigations should further explore the possibility of using attenuated oral recombinant bacteria as vaccine vectors that induce specific CD8+ T cell responses. Such vaccine-induced immune responses are critical for prevention or control of a number of pathogens such HIV.

## Methods

### Bacterial strains and culture conditions

Competent *Escherichia coli *SCS110 cells (Stratagene, USA) were used in cloning and genetic manipulations. An auxotroph, Δ*aro*C *Salmonella enterica *serovar Typhimurium mutant vaccine strain (TML-MD58) (Microscience Pty Ltd, UK) was used as an attenuated vaccine for the expression of GFP. The strain has a deletion in the *aro*C gene, which encodes chorismate synthase, an enzyme necessary for the biosynthesis of aromatic compounds, tryptophan, tyrosine, phenylalanine, para-aminobenzoic acid and 2,3-dihydroxybenzoate [37]. The bacteria were grown in 2YT media supplemented, where necessary, with ampicillin and aromatic amino acids (tryptophan, tyrosine, phenylalanine, para-aminobenzoic acid and 2,3-dihydroxybenzoate) as previously described [37,38].

### Construction of a high-level GFP expression cassette

Unless stated otherwise, DNA manipulations were performed using standard recombinant DNA methods [38]. A recombinant plasmid, designated pGEM+GFP, was constructed. The *gfp *gene was amplified using GFP2 (forward), 5'-ATG GCG CCA AAG ACT CCG GCT CCG-3' and GR (reverse), 5'- AAG CTT ATT TGT ATA GTT CAT CCA TGC-3') synthetic oligonucleotides as primers. The primers were rationally designed so that GR could have a preferred gram-negative bacterial stop codon, 5'-TAAG-3' at its end and that after cloning of the *gfp *PCR product in pGEM-Teasy (Promega, USA), there could be a second stop codon, TAAT, one codon downstream of TAAG. The primer GFP2 was designed so that the *gfp *gene to be amplified by polymerase chain reaction could be in-frame with the 5' domain (first 40 codons) of *β*-galactosidase *α*-gene in pGEM-Teasy vector. Restriction site for *Nar *I, 5'-GGCGCC-3', was incorporated in the GFP2 primer. The two primers had few base mismatches with their respective target DNA sequences in *gfp *template.

The polymerase chain reaction for amplification of *gfp *was conducted in a 50 μl volume with 4.5 units AmpliTaq Gold™ DNA polymerase (Applied Biosystems), 1× PCR buffer, 1.5 μM of each primers (GFP2 and GR), 0.2 mM deoxynucleotide triphosphates, 1.5 mM magnesium chloride and 10 ng of PEHAOGFP plasmid (provided by Dr W Bourn, University of Cape Town). The PCR cycling conditions were as follows: 1 cycle of 95°C for 5 min, 5 cycles of 95°C for 45 s, 55°C for 30 s, 72°C for 2 min, 25 cycles of 95°C for 45 s, 64°C for 30 s, 72°C for 2 min, and a final extension of 72°C for 7 min. Analysis of the *gfp *amplicon aliquot (5 μl) was done by agarose gel electrophoresis. An aliquot (1 μl) of remaining amplicon was ligated into a linearized pGEM-Teasy (Promega, USA) according to manufacturer's recommendations. The ligation reaction was used in the genetic transformation of competent *E. coli *SCS110 cells using the heat-shock method. The recombinant SCS110 clones harbouring the recombinant plasmid (pGEM+GFP) were screened for presence of *gfp *fragment and its orientation by blue-white screening procedure and UV-illumination. The white and fluorescing (candidate) clones were cultured using standard protocols. To investigate the presence of the recombinant *gfp *gene in plasmids, restriction mapping was performed initially with *Eco*R1 followed by double digestion with *Nar*I and *Hind*III. The *gfp *gene in the candidate pGEM+GFP plasmid was sequenced.

### Preparation of Δ*aroC Salmonella enterica *serovar Typhimurium expressing GFP

To investigate the expression of recombinant GFP, pGEM+GFP and pGEM (negative control) plasmids were used in the genetic transformation of competent *aroC Salmonella enterica *serovar Typhimurium mutant by a standard heat-shock method [33]. The agar plates were incubated overnight and fluorescence of colonies viewed under UV light the following morning. Single colonies were cultured in 100 ml 2 YT liquid broth with ampicillin (100 μg/ml). To determine the expression of GFP by the recombinant *Salmonella*, total bacterial protein was extracted from each culture, separated by a standard 12% sodium dodecyl sulphate-polyacrylamide gel electrophoresis (SDS-PAGE) and visualized in the gel by Coomassie blue staining. A standard Western blotting was performed to identify and to confirm the specificity and integrity of the GFP antigen band seen on the SDS-PAGE after Coomassie blue staining. A mixture of two anti-GFP mouse monoclonal antibodies (Clones 7.1 and 13.1) (Roche Diagnostics) was used as a primary antibody (diluted at 1.1000). Goat-anti-mouse immunoglobulins conjugated to horseradish peroxidase (Biorad), diluted at 1.1000 were used as secondary antibody. The immunoblot was visualized by enhanced chemiluminescence (Roche Diagnostics) and autoradiography according to manufacturer's recommendations

### Vaccination of mice and preparation of splenocytes

To prepare vaccine stocks, a single colony of recombinant *Salmonella *was inoculated into 200 ml of 2 YT liquid media supplemented with ampicillin (100 ug/ml), and aromatic amino acids (1×) and grown at 37°C with strong aeration. The bacterial cells were harvested in the logarithmic phase (OD_600 _= 0.8–1.0) by centrifugation at 3000 rpm for 5 mins, washed once with equal volume of phosphate buffered saline (PBS, pH 7.4) and suspended in PBS with 15% glycerol. The cultures were stored in aliquots at -80°C until vaccination. The bacterial count in the vaccine stocks was determined by plating of serial dilutions. The vaccines were designated AroC+GFP (a recombinant *aro*C *Salmonella enterica *serovar Typhimurium mutant expressing GFP) and AroC+pGEM (a recombinant control *aro*C *Salmonella enterica *serovar Typhimurium harbouring an empty plasmid, pGEM-Teasy).

All animal procedures were approved by the University of Cape Town Animal Ethics Committee. Female H-2^d ^BALB/c mice (8–10 weeks old; and five per group) were purchased from South Africa Vaccine Producers Pty Ltd (Johannesburg, South Africa), housed at the University of Cape Town Animal Unit and allowed to adapt for a minimum of 10 days before vaccinations. Groups of female BALB/c mice were inoculated by intragastric gavage with 10e8 colony forming units (CFUs)/mouse of either *Salmonella *vaccine (AroC+GFP) or negative control (AroC+pGEM) on Days 0, 28 and 56. Mice were sacrifice on Day 84 and spleens were pooled for each group. The spleens were meshed using a rubber stopper and metal grid (Sigma) placed in a petri dish to generate a single cell suspension in RPMI 1640 medium (Invitrogen, USA). The cell suspension was transferred to a 50 ml conical centrifuge tube. The volume was made up to 50 ml with RPMI 1640 medium. The cell suspension was centrifuged at 1500 rpm for 5 minutes to pellet the cells. The pellet was re-suspended in 50 ml of RPMI 1640 medium and centrifuged as before. The pellet was then washed twice with 50 ml of RPMI 1640 medium. The cells were re-suspended in 50 ml R10 medium (RPMI 1640, 10% heat-inactivated fetal calf serum, a mixture of pernicillin and streptomycin (Invitrogen, USA), and 15 mM 2-mercaptoethanol (Sigma, USA)). A single cell suspension of splenocytes was prepared and red cells were lysed using erythrocyte lysing buffer (0.15 M NH_4_Cl, 10 mM KHCO_3_, 0.1 mM Na_2_EDTA) for 1 min at room temperature. To count the cells and determine viability, 1/10 dilution of the suspension was made in Trypan Blue and Neubauer counting chamber used. Cell concentration in suspension was calculated and adjusted to an appropriate concentration. For use in ELISPOT assay, the splenocytes were adjusted to a concentration of 5 × 10e6 cells per ml and 100 ul of this stock was added to a single well which contained 100 ul of the stimulant. For use in CBA assay, the splenocytes were adjusted to a concentration of 15 × 10e6 cells per ml and 100 ul of this stock was added to a single well which contained 100 ul of the stimulant.

### IFN-γ and IL-4 ELISPOT assays

The IFN-γ and IL-4 ELISPOT kits (BD Pharmingen) were used according to manufacturer's recommendations. Splenocytes were plated in triplicate at 0.5 × 10e6 cells/well in a final volume of 200 μl of R10 medium (RPMI-1640 with 10% heat-inactivated fetal calf serum, 15 mM β-mercaptoethanol, 100 U penicillin per ml, and 100 μg streptomycin) either alone or with stimulants at 4 μg/ml. The stimulants used assays were media (no peptide), GFP H-2K^d ^binding peptide (HYLSTQSAL), full-length GFP, *Salmonella *lipopolysaccharide (LPS) (at a final concentration of 0.5 μg/ml). After incubation for 24 hrs (IFN-γ ELISPOT assay) or 48 hr (IL-4 ELISPOT assay), the plates were processed to detect IFN-γ- or IL-4-spot-forming units (SFUs) using Nova Red substrate (Vector Laboratories, UK) according to the kit instructions. Spots were counted using a CTL Analyzer (Cellular Technology, OH, USA) and ImmunoSpot Version 3.2 software (Cellular Technology OH, USA). The mean number of spots ± SD in triplicate wells was calculated and expressed as SFUs/10e6 splenocytes. Differences in immune responses between vaccine groups were analyzed by the two-sample t-test.

### Cytometric Bead Array (CBA) assay

Splenocytes at a concentration of 1.5 × 10e6 per 200 ul R10 culture medium (RPMI-1640 with 10% heat inactivated fetal calf serum, 100 U penicillin per ml, and 100 μg streptomycin) were cultured alone or with the individual stimulants as in the ELISPOT assay. CD8+ Tc1 (IFN-γ) and Tc2 (IL-4) cytokines secreted by the splenocytes were quantified using a mouse Th1/Th2 cytokine cytometric bead array (CBA) assay (BD Biosciences kit) and flow cytometry analysis according to manufacturer's instructions. Results were expressed as pg cytokine per 1 × 10e6 splenocytes. Differences in immune responses between vaccine groups were analyzed by the two-sample t-test.

## List of abbreviations

CBA: cytometric bead array; Con A: Concanavalin A; ELISPOT: Enzyme-linked immunospot; GFP: Green fluorescent protein; IFN-γ: interferon-gamma; LPS: lipopolysaccharide; IL-4: interleukin 4; SDS-PAGE: sodium dodecyl sulphate-polyacrylamide gel electrophoresis; SFUs: Spot-forming units; 2YT: 2× Yeast Tryptone.

## Competing interests

The authors declare that they have no competing interests.

## Authors' contributions

NC, WB, AW and EGS designed the experiment. NC performed all the experiments. NC, WB, AW and EGS all participated in the writing of the manuscript. All the authors read and approved the manuscript.
